# Editorialets

**Published:** 1907-10

**Authors:** 


### Editorialets.

TEXAS PHYSICIANS are urged to attend the Hot Springs meeting of the Southwestern Medical Association, October 7, 8 and 9. Those who desire to go should at once write Dr. Bacon Saunders, at Fort Worth, or Dr. E. H. Cary, Dallas, who will secure sleepers for them. Rosser is president. He will make a fine address, you may be sure, and a splendid program has been prepared.

THE MEDICAL ASSOCIATION of the Southwest will hold its annual meeting at Hot Springs, Ark., October 8, 9, 10, inst., Dr. F. H. Clark, El Reno, Okla., Secretary. A splendid program is prepared. "Our own" Dr. C. M. Rosser (Dallas), is President. Go. Special rates.

---

Dr. Geo. R. Tabor, the distinguished Texas sanitarian and late State Health Officer and Surgeon General of Texas, has removed to Dallas, Texas, and entered the general practice.

---

The Manor Sanitarium.—A commodious and well furnished and equipped building, with ample grounds and attractive surroundings, is offered for lease or sale. Correspondence solicited.

A. K. Anderson,
Jno. E. Hill,
John E. Sellstrom,
For Executive Board.

Manor, Travis County, Texas.

---

Dr. Robert Dinwiddie has been appointed by the mayor of San Antonio bacteriologist to the local Board of Health.

---

Dr. J. B. Shelmire, of Dallas, the well known and very popular dermatologist, attended as delegate the recent International Congress on Dermatology, in New York, and remaining over several weeks took a special course in his specialty.

---

Dr. Thos. R. Pettway, Austin, Texas, has been appointed by the Governor physician to the deaf-mute State institute.

